# Middle-aged and older populations with different subtypes and definitions of metabolic syndrome face different future cardiovascular disease risks: results from a comparison of two Chinese definitions

**DOI:** 10.1186/s12986-026-01095-3

**Published:** 2026-02-18

**Authors:** Hong Yang, Yunda Huang, Guihua Jiang, Hang Guo, Zhiping Duan

**Affiliations:** 1 Department of Geriatrics, The Third People’s Hospital of Yunnan Province, The Second Affiliated Hospital of Dali University, 292 Beijing Road, Kunming, 650011 unnan Province China; 2 Geriatrics Department, The People’s Hospital of Mengzi, Honghe, China

**Keywords:** Metabolic syndrome, Subtypes, Cardiovascular disease, Charls

## Abstract

**Background:**

Differences in the association between metabolic syndrome (MetS) and cardiovascular disease (CVD) across definitions and subtypes are unknown. The aim of this study was to investigate the differences between the associations of MetS defined by the Chinese Diabetes Society (CDS) and the Writing Group of 2024 Chinese Guidelines for the Management of Hypertension (WCGH) with CVD.

**Methods:**

This cohort study included participants aged ≥ 40 years without cardiovascular disease from the 2011 survey of the China Health and Retirement Longitudinal Study. Individuals meeting different definitions of MetS based on criteria from the CDS and the WCGH were identified and followed up until 2020. Cox proportional risk models were used to analyze the association between different definitions and subtypes of MetS with CVD, and the predictive performance of the models was compared using the area under the curve (AUC) of the time-dependent receiver operating characteristic curve, integrated discrimination improvement (IDI), and net reclassification improvement (NRI).

**Results:**

Both CDS and WCGH based MetS were significantly associated with increased CVD risk, with hazard ratios (HRs) and 95% confidence intervals (CIs) of 1.51 (1.38 ~ 1.66) and 1.81 (1.66 ~ 1.99), respectively, both *P* < 0.001. 7 of the 16 subtypes of MetS based on CDS were not associated with CVD; all 5 subtypes of MetS based on WCGH were significantly associated with an increased risk of CVD. When participants were grouped based on meeting two definitions, compared with the CDS-WCGH- group, CVD risk increased most significantly in the CDS-WCGH + group (HR and 95%CI: 2.57 [1.94 ~ 3.39], *P* < 0.001), showed a significant increase in the CDS + WCGH + group (HR and 95%CI: 1.66 [1.51 ~ 1.83], *P* < 0.001), and showed no significant increase in the CDS + WCGH- group (HR and 95%CI: 0.93 [0.7 ~ 1.23], *P* = 0.606). The AUC of WCGH was higher than that of CDS at all time points. IDI and NRI analyses showed that the WCGH standard demonstrated significant improvements in risk reclassification and identification compared to CDS.

**Conclusions:**

The association between MetS and CVD depends on the definition criteria and specific component combinations employed. The WCGH definition, which integrates diagnostic criteria for dyslipidemia, has been demonstrated to be more robust than the CDS.

**Graphical abstract:**

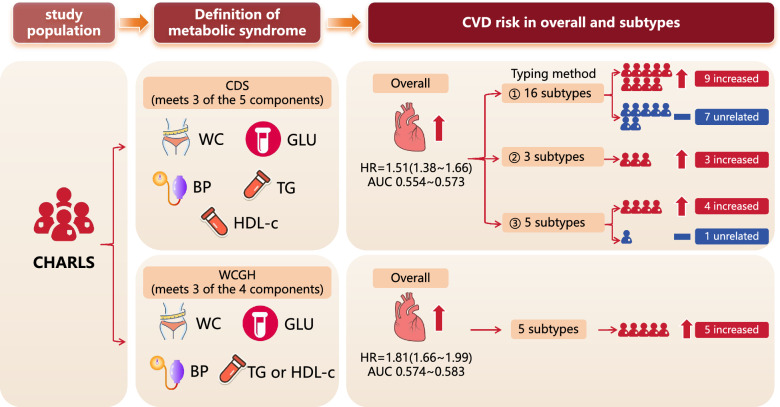

**Supplementary Information:**

The online version contains supplementary material available at 10.1186/s12986-026-01095-3.

## Background

Cardiovascular Disease (CVD), which mainly consists of ischemic heart disease and stroke, remains by far one of the leading causes of premature death and disability worldwide [1]. According to the Global Burden of Disease, the global burden of CVD has shown a steady upward trend in recent years, with the number of people with the disease increasing from 271 million in 1990 to 523 million in 2019, and the number of deaths increasing from 12.1 million to 18.6 million [2]. In China, the disease burden of CVD is equally severe, accounting for more than 40% of deaths in China, two-thirds of which are ischemic heart disease and stroke [3, 4]. The United Nations and the Chinese government have developed programs to reduce the prevalence and mortality of CVD [5, 6], and early detection and improvement of CVD risk factors is a very important part of these programs.

Metabolic syndrome (MetS) is a group of metabolic abnormalities characterized by a combination of abdominal obesity, elevated blood pressure, insulin resistance, and dyslipidemia [7]. The components of MetS contribute to the development and progression of CVD through mechanisms such as microangiopathy, atherosclerosis, cardiac overload, and myocardial injury [8–10]. A meta-analysis incorporating 951,083 participants primarily from North America, Europe, and Asia revealed that individuals with MetS had a 135% increased risk of CVD, a 99% increased risk of myocardial infarction, a 127% increased risk of stroke, and a 140% increased risk of cardiovascular mortality [11].

Although MetS has been shown to be associated with an increased risk of CVD [12], individuals with the same diagnosis of MetS may have very different CVD risks due to inconsistent associations of the components of MetS with CVD [13–15]. This is particularly noteworthy in specific populations; for instance, in Chinese patients with type 2 diabetes mellitus living in Taiwan, hypertension has been identified as the most significant component of MetS associated with ischemic heart disease [16]. The traditional dichotomous classification of MetS does not distinguish between these differences, so it is necessary to categorize MetS into multiple subtypes according to composition and to explore whether the risk of CVD differs among these subtypes. Meanwhile, since the definitions of MetS recommended by different organizations differ in the combination of diagnostic thresholds and components [17–21], analyzing the differences between MetS based on different definitions will facilitate standardized management of MetS. There are 2 widely used definitions of MetS for the Chinese population, which are recommended by the Chinese Diabetes Society (CDS) [20] and the Writing Group of 2024 Chinese Guidelines for the Management of Hypertension (WCGH) [21], and they differ somewhat in their composition. However, no studies have systematically compared the predictive value of these two Chinese-specific MetS definitions and their subtypes for future CVD risk. Therefore, the primary objectives of this study were (1) to evaluate and compare the strength of association between MetS, as defined by CDS and WCGH, and the incidence of CVD; and (2) to assess the predictive performance of different MetS subtypes for CVD outcomes, aiming to provide evidence for optimizing MetS diagnostic criteria and improving risk stratification in clinical practice.

## Methods

### Study population

The population for this longitudinal study was drawn from the CHARLS 2011–2020, a nationally representative longitudinal survey that collects health and economic data on the middle-aged and older populations in the Chinese community. CHARLS employs a multistage probability sampling design covering 28 provinces, 150 counties and districts, and 450 villages and communities across mainland China. Data are collected through computer-assisted personal interviews and physical examinations. The baseline survey was conducted in 2011 with follow-ups in 2013, 2015, 2018, and 2020, and detailed information about CHARLS has been reported previously [22].

**Fig. 1:**
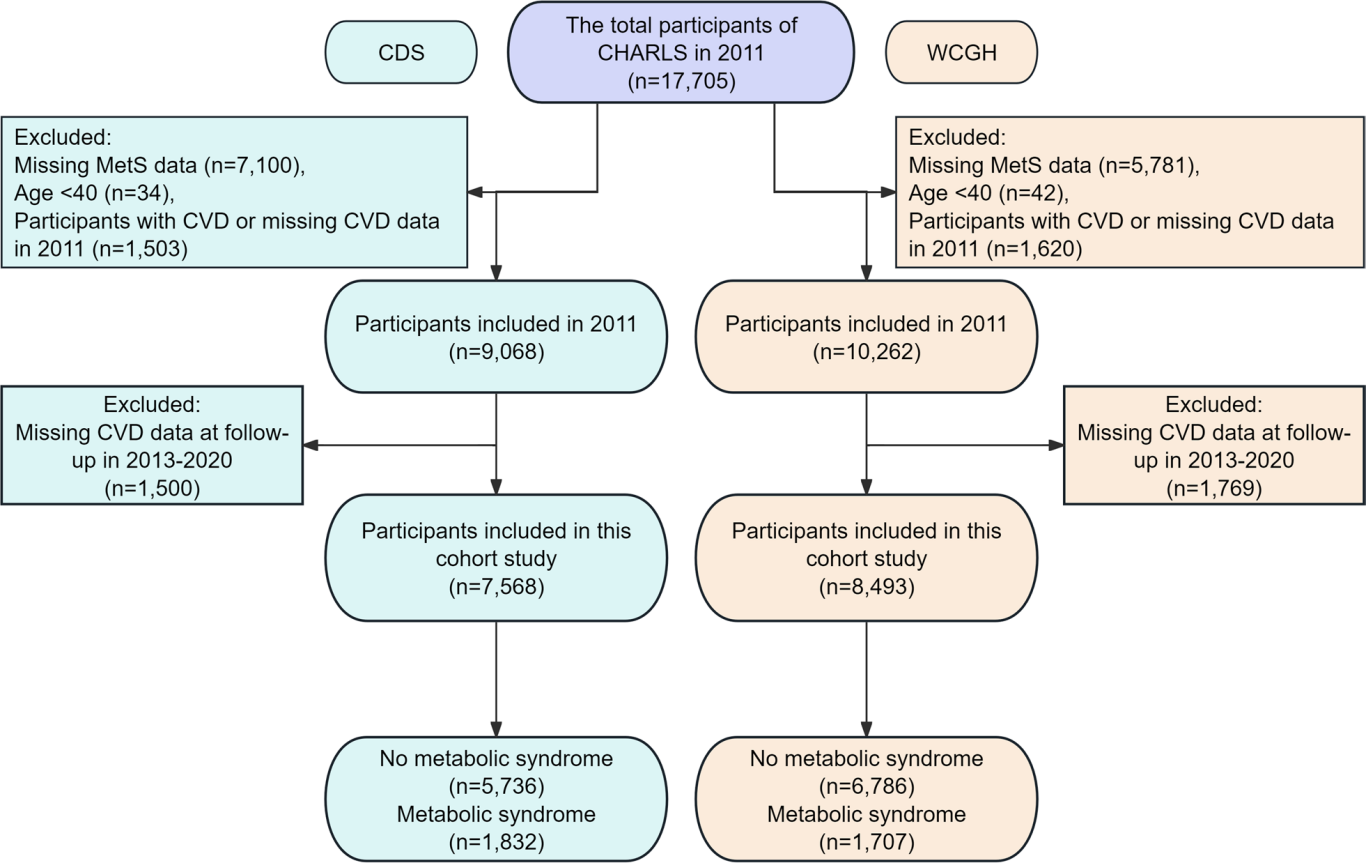
Flowchart of participant screening.. CDS, Chinese Diabetes Society; WCGH, Writing Group of 2024 Chinese Guidelines for the Management of Hypertension; MetS, metabolic syndrome; CVD, cardiovascular disease

We developed a homogenization process for screening appropriate participants, and based on the 2 definitions of MetS, we created 2 cohorts with some differences as study populations. Specifically, we excluded the following populations from the baseline survey in 2011: (1) missing MetS data (n1 = 7,100, n2 = 5,781), (2) age < 40 (n1 = 34, n2 = 42), (3) participants with CVD or missing CVD data in 2011 (n1 = 1,503, n2 = 1,620). On this basis, those who were lost to follow-up or had missing CVD data during the 2013–2020 follow-up were further excluded (n1 = 1,500, n2 = 1,769). n1 and n2 represent the number of people based on MetS definitions CDS and WCGH, respectively. Ultimately, the study population included 7,568 based on CDS and 8,493 based on WCGH (Fig. 1).

The CHARLS study was approved by the Biomedical Ethics Committee of Peking University (approval number: IRB00001052-11015), and informed consent was obtained from each respondent prior to their participation in the survey [22]. The study was conducted in accordance with the Strengthening the Reporting of Observational Studies in Epidemiology guideline recommendations related to cohort studies [23].

### Assessment of MetS

We used 2 criteria to define MetS, and they were proposed by the CDS and the WCGH [20, 21]. The definition based on the CDS is the presence of ≥ 3 of the following [20]: (1) abdominal obesity (AO): waist circumference ≥ 90 cm in males and ≥ 85 cm in females, (2) high glucose (GLU): fasting plasma glucose (FPG) ≥ 6.1 mmol/L or 2-h plasma glucose (PG) ≥ 7.8 mmol/L after oral glucose tolerance test (OGTT), or diabetes that has been diagnosed and treated, (3) high blood pressure (BP): blood pressure ≥ 130/85 mmHg or confirmed and treated hypertension, (4) fasting triglyceride (TG) ≥ 150 mg/dl, (5) fasting high density lipoprotein cholesterol (HDL-c) < 40 mg/dl.

Definition based on WCGH combined TG and HDL-c into a single risk factor [21]. Specifically, MetS was defined when ≥ 3 of the following 4 were present: (1) AO: waist circumference ≥ 90 cm in males and ≥ 85 cm in females, (2) high GLU: FPG ≥ 6.1 mmol/L or 2⁃h PG ≥ 7.8 mmol/L after OGTT, or diabetes that has been diagnosed and treated, (3) high BP: blood pressure ≥ 130/85 mmHg or confirmed and treated hypertension, (4) dyslipidemia: fasting TG ≥ 150 mg/dl, fasting HDL-c < 40 mg/dl, or diagnosed and treated with medications for dyslipidemia.

Participants' waist circumference was measured using a soft tape measure. Blood samples were collected by Chinese Center for Disease Control and Prevention staff, and fasting status was recorded. HbA1c was measured using the boronate affinity HPLC method, while TG, TC, HDL-C, LDL-C, and FPG were determined via enzymatic colorimetric tests. Blood pressure was measured using an Omron™ HEM-7112 (Omron Co., Ltd., Dalian, China) electronic sphygmomanometer for a total of 3 measurements at 45-s intervals. Interviewers obtained information on participants' previous hypertension, diabetes, and dyslipidemia by questionnaire.

### Assessment of CVD

Referring to previous studies [24, 25], CVD was defined as having a heart disease (HD) and/or stroke. In CHARLS, a questionnaire was used to determine whether participants had HD or stroke. Trained interviewers asked participants, “Have you been diagnosed with a heart attack, coronary heart disease, angina, congestive heart failure, or other heart problems by a doctor?” Participants answering “yes” were considered to have HD. Participants were considered to have had a stroke if they answered “yes” to the question, “Have you been diagnosed with a stroke by a doctor?” Therefore, participants were considered to have CVD if they answered “yes” to either of these two questions. The CVD onset period was defined as the interval between the last interview and the one in which the CVD was first recorded.

### Covariates

Covariates included in this study were demographic information, lifestyle habits, and chronic disease status associated with CVD. Demographic information included gender, age, education, marriage, and place of residence. Lifestyle habits included smoking status and alcohol intake. To control for potential confounding factors, we included chronic lung disease and chronic kidney disease as covariates, both of which are associated with CVD. This information was obtained through questionnaires.

### Statistical analysis

To compare the association between subtypes of MetS and CVD based on the two definitions, all analyses were performed separately in the two cohorts. In the cohort based on CDS, we used three methods to categorize MetS. The first method was to categorize MetS based on different combinations of its components, categorizing MetS into 16 subtypes. However, using so many subtypes to manage the disease and distinguish their nuances is very impractical, and to make it more convenient for practical applications, we created 2 additional new methods to recombine these subtypes. The 2nd typing method is to categorize MetS into 3 subtypes based on the number of components that match 3, 4, and 5 components of MetS, respectively. Considering that both TG and HDL-c are lipid-metabolizing components, the 3rd typing method we used was based on the 2nd method and further categorized MetS into 5 subtypes based on whether or not it contained both high TG and low HDL-c.

In the cohort based on WCGH, we categorized MetS into 5 subtypes based on different combinations of constituents, where 4 subtypes were each consistent with 3 different constituents, and the 5th subtype was consistent with all 4 constituents.

Continuous variables are expressed as mean ± standard deviation or median and interquartile range, and categorical variables are expressed as counts and percentages. We compared the baseline characteristics of participants with and without MetS in the two cohorts separately. To further understand the differences between different definitions of MetS, we also compared baseline characteristics between participants with MetS based on two definitions. All baseline characteristics were analyzed using t-tests, chi-square tests, or nonparametric tests. Hazard ratios (HRs) and 95% confidence intervals (CIs) for the components and subtypes of MetS were estimated using the Cox proportional risk model. We used the Schoenfeld residual test to verify the proportional risk hypothesis, and the p-value of the Schoenfeld residual test for all Cox models was > 0.05. The cumulative risk of MetS and its subtypes over time was assessed using Kaplan–Meier cumulative risk curves and log-rank tests. Subgroup analyses were used to assess differences in subtypes of MetS across gender and age levels. To correct for potential bias due to sample selection, we estimated propensity scores and inverse probability weights for each sample using a logistic regression model and analysed associations between different definitions and subtypes of MetS with CVD using a Cox model weighted by inverse probability. In addition, we used the area under the curve (AUC) and 95%CI of time dependent receiver operating characteristic curve (ROC) to assess the predictive power of differently defined MetS for CVD.

To directly compare differences between the two definitions of MetS, we divided the population into four groups at baseline based on the two MetS definitions: the CDS- WCGH- group, the CDS + WCGH- group, the CDS- WCGH + group, and the CDS + WCGH + group. Logistic regression and Cox models were used to compare the cross-sectional and longitudinal associations between MetS defined by different criteria and CVD. Integrated discrimination improvement (IDI) and net reclassification improvement (NRI) were employed to analyze the differences between the two MetS definitions. All analyses were performed with CVD, stroke, and HD as outcome events, respectively.

All data analyses were performed using statistical software Stata/MP version 17.0 and the “timeROC” and “survIDINRI” packages in R version 4.4.1. Graphical representations were created using the ‘ggplot2’ package in R and the “matplotlib” package in Python version 3.12. The level of statistical significance was set at *P* < 0.05 (two-sided).

## Results

### Baseline characteristics of participants

In baseline characteristics based on CDS, the mean age of the participants was 57.9 ± 8.9, and 54.7% were female. Participants with MetS were more likely to be female, older, more educated, and live in towns, less likely to smoke, consume alcohol, and have kidney disease compared with those without MetS, all *P* < 0.05. They also differed significantly in indicators of obesity, blood pressure, blood glucose, and lipids, all *P* < 0.05 (Table 1). MetS, metabolic syndrome; BMI, body mass index; GLU, glucose; HBA1c, glycosylated hemoglobin; SBP, systolic blood pressure; DBP, diastolic blood pressure; CHO, cholesterol; HDL-c, high density lipoprotein cholesterol; LDL-c, low density lipoprotein cholesterol; TG, triglycerides. SD, standard deviation; IQR, interquartile range

**Table 1:** Baseline Characteristics of Participants

	CDS				WCGH				
	Total (n = 7,568)	No MetS (n = 5,736)	MetS (n = 1,832)	*P*	Total (n = 8,493)	No MetS (n = 6,786)	MetS (n = 1,707)	*P*	P*
**Gender**, n (%)				< 0.001				< 0.001	0.667
Female	4,135 (54.7)	3,063 (53.4)	1,072 (58.5)		4,545 (53.5)	3,534 (52.1)	1,011 (59.2)		
Male	3,428 (45.3)	2,668 (46.6)	760 (41.5)		3,943 (46.5)	3,247 (47.9)	696 (40.8)		
**Age**, year, mean ± SD	57.9 ± 8.9	57.6 ± 8.9	58.8 ± 8.8	< 0.001	57.6 ± 8.8	57.2 ± 8.8	59.3 ± 8.7	< 0.001	0.071
**Education**, n (%)				0.033				0.074	0.988
Primary school or below	5,184 (68.5)	3,955 (69)	1,229 (67.1)		5,769 (67.9)	4,628 (68.2)	1,141 (66.8)		
Middle school	1,582 (20.9)	1,203 (21)	379 (20.7)		1,809 (21.3)	1,453 (21.4)	356 (20.9)		
High school or above	802 (10.6)	578 (10.1)	224 (12.2)		915 (10.8)	705 (10.4)	210 (12.3)		
**Marital**, n (%)				0.741				0.145	0.487
Other	704 (9.3)	530 (9.2)	174 (9.5)		788 (9.3)	614 (9)	174 (10.2)		
Married	6,864 (90.7)	5,206 (90.8)	1,658 (90.5)		7,705 (90.7)	6,172 (91)	1,533 (89.8)		
**Residence**, n (%)				< 0.001				< 0.001	0.268
Urban areas	1,194 (15.8)	810 (14.1)	384 (21)		1,356 (16.0)	972 (14.3)	384 (22.5)		
Rural areas	6,366 (84.2)	4,919 (85.9)	1,447 (79)		7,127 (84.0)	5,805 (85.7)	1,322 (77.5)		
**Smoking status**, n (%)				< 0.001				< 0.001	0.965
Never smoked	4,732 (63.2)	3,519 (62.1)	1,213 (66.7)		5,247 (62.3)	4,110 (61.2)	1,137 (66.9)		
Former smoker	547 (7.3)	386 (6.8)	161 (8.9)		612 (7.3)	459 (6.8)	153 (9)		
Current smoker	2,210 (29.5)	1,766 (31.1)	4,44 (24.4)		2,558 (30.4)	2,149 (32)	409 (24.1)		
**Alcohol consumption**, n (%)				< 0.001				< 0.001	0.928
Never or rarely	5,007 (66.2)	3,728 (65)	1,279 (69.9)		5,590 (65.8)	4,389 (64.7)	1,201 (70.4)		
Less than once a month	617 (8.2)	477 (8.3)	140 (7.6)		721 (8.5)	595 (8.8)	126 (7.4)		
More than once a month	1,941 (25.7)	1,529 (26.7)	412 (22.5)		2,179 (25.7)	1,800 (26.5)	379 (22.2)		
**Chronic diseases**, n (%)									
Chronic lung diseases	657 (8.7)	506 (8.8)	151 (8.3)	0.443	739 (8.7)	589 (8.7)	150 (8.8)	0.883	0.557
Kidney disease	444 (5.9)	354 (6.2)	90 (4.9)	0.046	488 (5.8)	404 (6)	84 (4.9)	0.1	0.993
Hypertension	2,683 (35.5)	1,531 (26.7)	1,152 (62.9)	< 0.001	2,718 (32.0)	1,487 (21.9)	1,231 (72.1)	< 0.001	< 0.001
Diabetes	1,061 (14.0)	377 (6.6)	684 (37.3)	< 0.001	1,066 (12.6)	398 (5.9)	668 (39.1)	< 0.001	0.272
Dyslipidemia	2,666 (35.7)	1,281 (22.7)	1,385 (76)	< 0.001	3,632 (43.0)	2,353 (34.9)	1,279 (75.4)	< 0.001	0.655
**Biomarkers**									
Waist circumference, cm, mean ± SD	83.8 ± 12.4	81.0 ± 11.7	92.3 ± 10.7	< 0.001	83.1 ± 12.3	80.5 ± 11.5	93.3 ± 9.9	< 0.001	0.004
BMI, kg/m^2^, mean ± SD	23.5 ± 3.8	22.6 ± 3.4	26.1 ± 3.7	< 0.001	23.3 ± 3.8	22.5 ± 3.3	26.4 ± 3.7	< 0.001	0.061
GLU, mg/dl, mean ± SD	108.1 ± 33.2	101.8 ± 23.4	128.3 ± 48.4	< 0.001	108.0 ± 32.9	101.9 ± 22.7	130.1 ± 50.4	< 0.001	0.294
HBA1c, %, mean ± SD	5.2 ± 0.8	5.1 ± 0.6	5.6 ± 1.1	< 0.001	5.2 ± 0.8	5.1 ± 0.6	5.6 ± 1.1	< 0.001	0.443
SBP, mmHg, mean ± SD	128.9 ± 20.7	125.6 ± 19.9	138.9 ± 20.0	< 0.001	127.1 ± 20.3	123.5 ± 19.0	141.1 ± 19.4	< 0.001	0.001
DBP, mmHg, mean ± SD	75.3 ± 11.9	73.6 ± 11.6	80.6 ± 11.5	< 0.001	74.5 ± 11.8	72.7 ± 11.2	81.7 ± 11.2	< 0.001	0.005
CHO, mg/dl, mean ± SD	193.3 ± 37.9	190.7 ± 35.9	201.8 ± 42.4	< 0.001	193.2 ± 37.9	190.6 ± 35.9	202.6 ± 43.2	< 0.001	0.578
HDL-c, mg/dl, mean ± SD	51.9 ± 15.1	55.7 ± 14.1	39.8 ± 11.3	< 0.001	51.9 ± 15.1	54.6 ± 14.7	42.1 ± 12.2	< 0.001	< 0.001
LDL-c, mg/dl, mean ± SD	116.7 ± 34.3	117.2 ± 32.5	115.2 ± 39.6	0.034	116.6 ± 34.3	116.2 ± 32.8	118.0 ± 39.4	0.079	0.043
TG, mg/dl, median (IQR)	101.8 (73.5, 146.7)	89.4 (67.3, 119.5)	184.1 (138.1, 252.9)	< 0.001**	101.8 (73.5, 146.0)	92.0 (68.1, 126.6)	167.3 (114.2, 235.9)	< 0.001**	< 0.001**

P*, Comparison between MetS groups for CDS and WCGH^**^, using the Mann–Whitney U test. MetS, metabolic syndrome; BMI, body mass index; GLU, glucose; HBA1c, glycosylated hemoglobin; SBP, systolic blood pressure; DBP, diastolic blood pressure; CHO, cholesterol; HDL-c, high density lipoprotein cholesterol; LDL-c, low density lipoprotein cholesterol; TG, triglycerides. SD, standard deviation; IQR, interquartile range

Among the baseline characteristics based on WCGH, the mean age of the participants was 57.6 ± 8.8, and 53.5% were female. Compared with no MetS, participants with MetS had non-significant differences in educational attainment, kidney disease, and low density lipoprotein cholesterol (LDL-c) levels, and other characteristics had similar trends as CDS (Table 1).

We further compared baseline characteristics between participants with the two definitions of MetS. The prevalence of MetS based on CDS and WCGH was 24.2% (1,832/7,568) and 20.1% (1,707/8,493), respectively. Compared with participants with CDS MetS, participants with WCGH MetS had higher systolic blood pressure (141.1 vs. 138.9 mmHg), diastolic blood pressure (81.7 vs. 80.6 mmHg), percent hypertension (72.1 vs. 62.9%), waist circumference (93.3 vs. 92.3 cm), HDL-c (42.1 vs. 39.8 mg/dl), and LDL-c (118 vs. 115.2 mg/dl), and had a lower TG (167.3 vs. 184.1 mg/dl) (Table 1).

### Association of components and subtypes of MetS with CVD based on CDS

A total of 2,115 (27.9%) participants had new-onset CVD during the 9-year observation period, including 722 (9.5%) strokes and 1,626 (21.5%) HD. The prevalence of CVD (37.1% vs. 25%), stroke (13.9% vs. 8.1%), and HD (27.6% vs. 19.5%) was higher in the MetS group than in the no MetS group. AO, high GLU, high BP, high TG, and dyslipidemia were significantly associated with increased risk of CVD, stroke, and HD, all *P* < 0.01. Low HDL-c was associated with increased risk of CVD (HR = 1.14 [1.03 ~ 1.27], *P* = 0.011) and stroke (HR = 1.25 [1.05 ~ 1.48], *P* = 0.013), but the association with HD was not significant (HR = 1.09 [0.97 ~ 1.23], *P* = 0.141) (Table 2). Among all five MetS components, high BP had the strongest associations with CVD (HR = 1.73 [1.57 ~ 1.9], *P* < 0.001), stroke (HR = 2.07 [1.75 ~ 2.44], *P* < 0.001), and HD (HR = 1.64 [1.48 ~ 1.83], *P* < 0.001) (Table 2). The MetS based on CDS significantly increased the risk of CVD (HR = 1.51 [1.38 ~ 1.66], *P* < 0.001), stroke (HR = 1.73 [1.48 ~ 2.02], *P* < 0.001), and HD (HR = 1.4 [1.26 ~ 1.56], *P* < 0.001) (Table 2). The results of the univariate analyses were similar to the multivariate (Table S1).

**Table 2:** Results of multifactorial Cox models of MetS and its components with CVD, stroke, and HD

	**CVD**			**Stroke**			**HD**		
	**Cases (%)**	**HR (95% CI)**	***P***	**Cases (%)**	**HR (95% CI)**	***P***	**Cases (%)**	**HR (95% CI)**	***P***
**CDS**									
AO	948 (34.6)	1.58 (1.44 ~ 1.74)	** < 0.001**	330 (12)	1.73 (1.47 ~ 2.03)	** < 0.001**	723 (26.4)	1.47 (1.32 ~ 1.63)	** < 0.001**
High GLU	702 (34)	1.32 (1.2 ~ 1.44)	** < 0.001**	267 (12.9)	1.49 (1.28 ~ 1.74)	** < 0.001**	526 (25.5)	1.26 (1.13 ~ 1.4)	** < 0.001**
High BP	1,262 (35.6)	1.73 (1.57 ~ 1.9)	** < 0.001**	474 (13.4)	2.07 (1.75 ~ 2.44)	** < 0.001**	952 (26.8)	1.64 (1.48 ~ 1.83)	** < 0.001**
High TG	544 (32.2)	1.25 (1.14 ~ 1.38)	** < 0.001**	200 (11.8)	1.41 (1.19 ~ 1.66)	** < 0.001**	405 (24)	1.17 (1.05 ~ 1.32)	**0.006**
Low HDL-c	478 (30.3)	1.14 (1.03 ~ 1.27)	**0.011**	176 (11.2)	1.25 (1.05 ~ 1.48)	**0.013**	356 (22.6)	1.09 (0.97 ~ 1.23)	0.141
Dyslipidemia	869 (32.9)	1.35 (1.23 ~ 1.47)	** < 0.001**	320 (12.1)	1.52 (1.31 ~ 1.77)	** < 0.001**	658 (24.9)	1.29 (1.16 ~ 1.42)	** < 0.001**
MetS	680 (37.1)	1.51 (1.38 ~ 1.66)	** < 0.001**	255 (13.9)	1.73 (1.48 ~ 2.02)	** < 0.001**	506 (27.6)	1.4 (1.26 ~ 1.56)	** < 0.001**
**WCGH**									
AO	980 (35)	1.69 (1.55 ~ 1.85)	** < 0.001**	339 (12.1)	1.85 (1.58 ~ 2.16)	** < 0.001**	749 (26.7)	1.58 (1.43 ~ 1.75)	** < 0.001**
High GLU	697 (34.1)	1.32 (1.2 ~ 1.44)	** < 0.001**	261 (12.8)	1.47 (1.26 ~ 1.71)	** < 0.001**	524 (25.6)	1.26 (1.13 ~ 1.4)	** < 0.001**
High BP	1,289 (36)	1.84 (1.69 ~ 2.02)	** < 0.001**	477 (13.3)	2.16 (1.85 ~ 2.53)	** < 0.001**	977 (27.3)	1.77 (1.6 ~ 1.96)	** < 0.001**
High TG	523 (32.2)	1.26 (1.14 ~ 1.39)	** < 0.001**	190 (11.7)	1.4 (1.18 ~ 1.66)	** < 0.001**	392 (24.2)	1.18 (1.05 ~ 1.33)	**0.004**
Low HDL-c	457 (30.2)	1.14 (1.03 ~ 1.27)	**0.013**	166 (11)	1.23 (1.03 ~ 1.47)	**0.02**	343 (22.7)	1.1 (0.97 ~ 1.24)	0.124
Dyslipidemia	881 (33.1)	1.36 (1.24 ~ 1.48)	** < 0.001**	315 (11.8)	1.48 (1.27 ~ 1.72)	** < 0.001**	674 (25.3)	1.31 (1.18 ~ 1.45)	** < 0.001**
MetS	697 (40.8)	1.81 (1.66 ~ 1.99)	** < 0.001**	260 (15.2)	2.06 (1.77 ~ 2.4)	** < 0.001**	524 (30.7)	1.69 (1.52 ~ 1.87)	** < 0.001**

MetS, metabolic syndrome; CVD, cardiovascular disease; HD, heart disease; HR, hazard ratio; CI, confidence interval; AO, abdominal obesity; GLU, glucose; BP, blood pressure; TG, triglycerides; HDL-c, high density lipoprotein cholesterolAdjusted: gender, age, education, marriage, residence, smoking, alcohol intake, chronic lung disease, kidney disease. When *P* < 0.05, it is marked in bold.

**Fig. 2:**
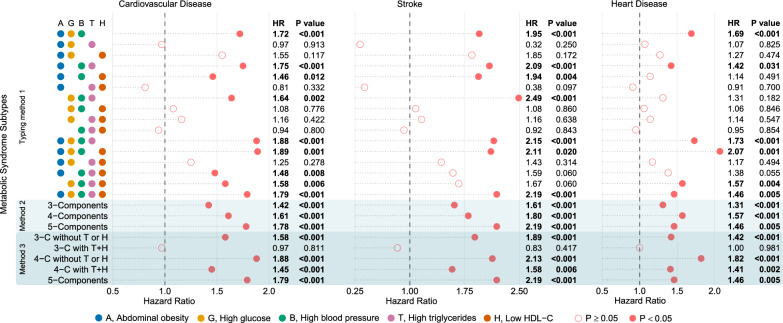
Results of multifactorial Cox models of subtypes of MetS with CVD, stroke, and HD. (CDS). The different coloured dots in the Y-axis labels represent each of the 5 components of CDS, and subtypes of metabolic syndrome are represented by combinations of dots. Pink dots represent hazard ratios and are indicated as hollow dots when P ≥ 0.05 and solid dots when P < 0.05.. Adjusted: gender, age, education, marriage, residence, smoking, alcohol intake, chronic lung disease, kidney disease

Figure 2 shows the results of multifactorial Cox models of subtypes of MetS based on CDS with CVD, stroke, and HD. Of the 16 subtypes in typing method 1, the subtypes most significantly associated with increased risk of CVD, stroke, and HD were AO + GLU + BP + HDL-c (HR = 1.89 [1.29 ~ 2.77], *P* = 0.001), GLU + BP + TG (HR = 2.49 [1.59 ~ 3.9], *P* < 0.001), and AO + GLU + BP + HDL-c (HR = 2.07 [1.36 ~ 3.13], *P* = 0.001), respectively. Of note, 7 subtypes were not associated with CVD, and the number of subtypes not associated with stroke and HD was 9 and 10, respectively, all *P* > 0.05.

All 3 subtypes were significantly associated with outcome events in analyses of typing method 2. The strongest associations with CVD (HR = 1.78 [1.44 ~ 2.22]) and stroke (HR = 2.19 [1.56 ~ 3.08]) were found for the MetS that met all 5 components; differently, the strongest associations were found for HD (HR = 1.57 [1.33 ~ 1.86]) for the MetS that met 4 components, all *P* < 0.001 (Fig. 2).

**Fig. 3:**
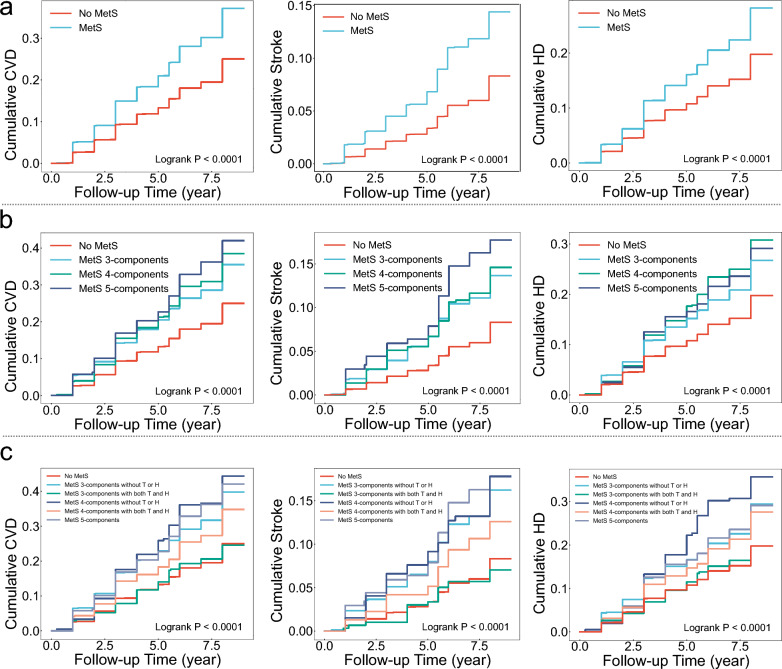
Kaplan–Meier cumulative risk curves for different subtypes of MetS with CVD, stroke, and HD. (CDS). **a**, Unsubtyped.. **b**, MetS was categorized into 3 subtypes by typing method 2. All subtypes significantly increase the risk of outcome events.. **c**, MetS was categorized into 5 subtypes by typing method 3. Subtypes that met 3 components and contained both high triglycerides and low high density lipoprotein cholesterol did not increase the risk of outcome events

**Fig. 4:**
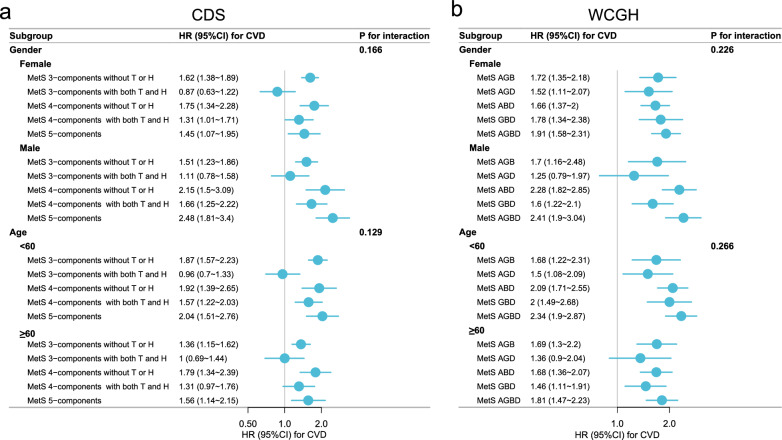
Subgroup forest plot of the association of MetS subtypes with CVD.. **a**, MetS based on CDS, which was categorized into 5 subtypes using typing method 3.. **b**, MetS based on WCGH, which was categorized into 5 subtypes based on different combinations of constituents. A, G, B, and D represent the four components of MetS: A, abdominal obesity; G, high glucose; B, high blood pressure; D, dyslipidemia. Different letter combinations denote corresponding MetS subtypes

Notably, in typing method 3, we found that subtype that met only 3 components and included TG + HDL-c were not at increased risk of CVD (HR = 0.97 [0.76 ~ 1.24], *P* = 0.811), stroke (HR = 0.83 [0.52 ~ 1.31], *P* = 0.417), or HD (HR = 1 [0.77 ~ 1.31], *P* = 0.981). All other 4 subtypes were significantly associated with outcome events, and the weakest associations with outcome events were found for the subtype that met the 4 components and included TG + HDL-c. The subtype that met 4 components and was without TG or HDL-c had the strongest associations with CVD (HR = 1.88 [1.52 ~ 2.33], *P* < 0.001) and HD (HR = 1.82 [1.43 ~ 2.32], *P* < 0.001); the strongest association with stroke (HR = 2.19 [1.56 ~ 3.08], *P* < 0.001) was the subtype that met the 5 components (Fig. 2). Univariate analyses (Table S2) and Kaplan–Meier cumulative risk curves (Fig. 3) gave similar results to the multivariate analyses. In subgroup analyses, this result did not differ across sex and age subgroups, with both *P* for interaction > 0.05 (Fig. 4a).

In the Cox model after inverse probability weighting, the association between MetS based on the CDS definition and CVD was similar to the pre-weighting results (Tables S3-S4).

### Association of components and subtypes of MetS with CVD based on WCGH

A total of 2,270 (26.7%) participants had new-onset CVD during the 9-year observation period, including 758 (8.9%) strokes and 1,750 (20.6%) HD. The prevalence of CVD (40.8% vs. 23.2%), stroke (15.2% vs. 7.3%), and HD (30.7% vs. 18.1%) was higher in the MetS group than in the no MetS group. All 4 components were significantly associated with an increased risk of outcome events, with high BP having the strongest associations with CVD (HR = 1.84 [1.69 ~ 2.02]), stroke (HR = 2.16 [1.85 ~ 2.53]), and HD (HR = 1.77 [1.6 ~ 1.96]), all *P* < 0.001 (Table 2). Similar to CDS, when dyslipidemia was subdivided into high TG and low HDL-c, the association between low HDL-c and HD was not significant (HR = 1.1 [0.97–1.24], *P* = 0.124). Compared to CDS, WCGH-based MetS was associated with higher HRs for CVD (HR = 1.81 [1.66 ~ 1.99]), stroke (HR = 2.06 [1.77 ~ 2.4]), and HD (HR = 1.69 [1.52 ~ 1.87]), all *P* < 0.001 (Table 2). The results of the univariate analyses were similar to the multivariate (Table S5).

**Fig. 5:**
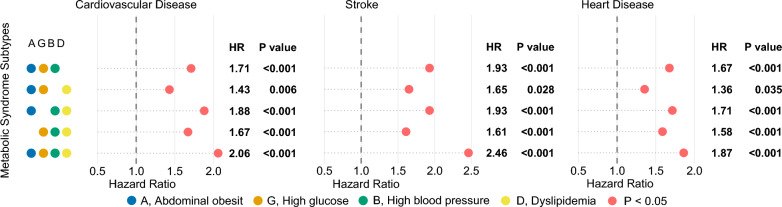
Results of multifactorial Cox models of subtypes of MetS with CVD, stroke, and HD. (WCGH). The different coloured dots in the Y-axis labels represent each of the 4 components of WCGH, and subtypes of metabolic syndrome are represented by combinations of dots. Pink solid dots represent hazard ratios, all P < 0.05.. Adjusted: gender, age, education, marriage, residence, smoking, alcohol intake, chronic lung disease, kidney disease

**Fig. 6:**
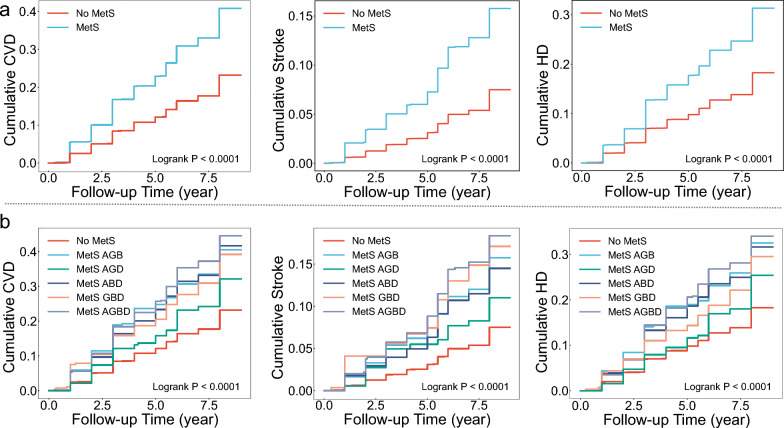
Kaplan–Meier cumulative risk curves for different subtypes of MetS with CVD, stroke, and HD. (WCGH). **a**, Unsubtyped.. **b**, MetS is categorized into 5 subtypes based on different combinations of components. A, G, B, and D represent the four components of MetS: A, abdominal obesity; G, high glucose; B, high blood pressure; D, dyslipidemia. Different letter combinations denote corresponding MetS subtypes. All subtypes significantly increase the risk of outcome events

Figure 5 shows the results of the multifactorial Cox model for subtypes of MetS based on WCGH, with all 5 subtypes associated with an increased risk of outcome events. The strongest associations were found between the subtype meeting all 4 components and CVD (HR = 2.06 [1.78 ~ 2.38]), stroke (HR = 2.46 [1.94 ~ 3.12]), and HD (HR = 1.87 [1.58 ~ 2.22]), all *P* < 0.001. The weakest associations were found between the subtype AO + GLU + dyslipidemia and CVD (HR = 1.43 [1.1 ~ 1.85], *P* = 0.006), stroke (HR = 1.65 [1.06 ~ 2.59], *P* = 0.028), and HD (HR = 1.36 [1.02 ~ 1.82], *P* = 0.035). Univariate analyses (Table S6) and Kaplan–Meier cumulative risk curves (Fig. 6) gave similar results to the multivariate analyses. In subgroup analyses, this result did not differ across sex and age subgroups, with both *P* for interaction > 0.05 (Fig. 4b).

In the Cox model after inverse probability weighting, the association between MetS based on the WCGH definition and CVD was similar to the pre-weighting results (Tables S7-S8).

### The time dependent ROC

**Fig. 7:**
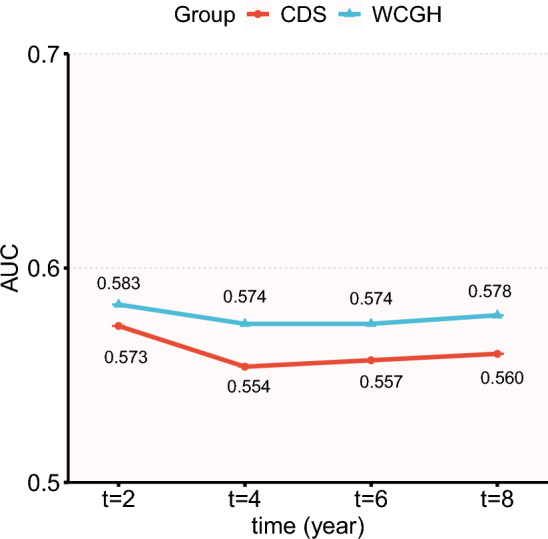
AUC for time dependent ROC. AUC, area under the curve; ROC, receiver operating characteristic curve

In the time-dependent ROC, the AUC for WCGH-based MetS (range: 0.574 ~ 0.583) was higher than that for CDS-based MetS (range: 0.554 ~ 0.573) at each time point (Fig. 7, Table S9).

### Association between MetS status grouped by two definitions and CVD

We excluded participants with missing MetS data (n = 7,327), age < 40 years (n = 32), and missing CVD data (n = 50) from the baseline analysis, finally including 10,296 participants in the cross-sectional analysis. Results from univariate (Table S10) and multivariate (Table S11) logistic regression models were generally consistent. After adjusting for all covariates, the CDS + WCGH- group showed no significant increase in CVD risk compared to the CDS- WCGH- group, odds ratio (OR) and 95%CI was 1.11 (0.76 ~ 1.61), *P* = 0.597. Both the CDS- WCGH + and CDS + WCGH + groups showed significantly increased CVD risk, with OR and 95% CI of 3.12 (2.17 ~ 4.5) and 2.23 (1.97 ~ 2.54), respectively, both *P* < 0.001 (Table S11).

After further excluding participants with CVD at baseline (n = 1394) and those with missing follow-up data (n = 1,471), a total of 7,431 participants were included in the longitudinal analysis. Results from univariate (Table S12) and multivariate (Table S13) Cox models were generally consistent. After adjusting for all covariates, the CDS + WCGH- group showed no significant increase in CVD risk compared with the CDS- WCGH- group, the HR and 95% CI were 0.93 (0.7 ~ 1.23), *P* = 0.606. Both the CDS- WCGH + and CDS + WCGH + groups showed significantly increased CVD risk, with HR and 95% CI of 2.57 (1.94 ~ 3.39) and 1.66 (1.51 ~ 1.83), respectively, both *P* < 0.001 (Table S13).

To further quantify the improvement in predictive accuracy offered by the WCGH definition, we calculated the NRI and IDI. For the outcome of CVD in cross-section analysis, the categorical and continuous NRI were 0.0352 (95%CI: 0.0203 ~ 0.0501, *P* < 0.001) and 0.4050 (95%CI: 0.3497 ~ 0.4603, *P* < 0.001), respectively. The IDI was 0.0052 (95%CI: 0.0036 ~ 0.0069, *P* < 0.001) (Table S14). In the longitudinal analysis, the categorical and continuous NRI were 0.021 (95% CI: 0.008 ~ 0.037, *P* < 0.001) and 0.171 (95%CI: 0.109 ~ 0.192, *P* < 0.001), respectively. The IDI was 0.009 (95%CI: 0.006 ~ 0.012, *P* < 0.001) (Table S15). This indicates that the use of the WCGH standard demonstrates significant improvement over the CDS standard in risk reclassification and identification.

## Discussion

This cohort study assessed the differences between 2 definitions of MetS that are widely used in China and found significant differences in the risk of CVD among participants with different definitions and subtypes of MetS. Overall, both CDS- and WCGH-based MetS were significantly associated with CVD. The association between MetS and CVD, as defined by CDS, was characterized by the following features: (1) 7 subtypes were not associated with CVD risk when typed according to combinations of components; (2) the subtype that met 3 components and contained both TG and HDL-c were not associated with CVD (HR = 0.97 [0.76 ~ 1.24], *P* = 0.811); and (3) the subtype that met 4 components and without TG or HDL-c was associated with CVD (HR = 1.88 [1.52 ~ 2.33], *P* < 0.001) and had the strongest association. In the MetS of WCGH, all 5 subtypes were significantly associated with an increased risk of CVD; this association was strongest in the subtype meeting the 4 components (HR = 2.06 [1.78 ~ 2.38]); it was weakest in the subtype with AO + GLU + dyslipidemia (HR = 1.43 [1.1 ~ 1.85], *P* = 0.006). These associations did not differ across gender and age. The results of the Cox model with inverse probability weighting are similar to the unweighted results. In the time dependent ROC, WCGH-based MetS had a higher AUC at each time point than CDS-based. When participants were grouped based on meeting both definitions, compared to the CDS-WCGH- group, the CVD risk in the CDS-WCGH + group and CDS + WCGH + group increased by 157% and 66%, respectively, while no significant increase was observed in the CDS + WCGH- group. IDI and NRI analyses showed that the WCGH standard demonstrated significant improvements in risk reclassification and identification compared to CDS.

In our study, the prevalence of MetS differed between definitions. In the meta-analysis by Li R et al. [26], when using the definition of MetS recommended by the International Diabetes Federation in 2005 (IDF) [19], the prevalence of MetS in mainland China was 24.5%, which was similar to that of the CDS, although both definitions differed in terms of cutpoints and combinations. Additionally, age and location also influence the prevalence of MetS. In a study by Lee-Ching Hwang et al. involving adults with a mean age of 44.7 years in Taiwan, China, the prevalence of MetS ranged from 14.3% to 16.4% depending on the diagnostic criteria used, which is significantly lower than our findings [27].

The risk of CVD varied between MetS definitions. In our study, MetS defined by WCGH resulted in an increased risk of CVD, stroke, and HD of 81%, 106%, and 69%, respectively; in the CDS, it was 51%, 73%, and 40%, respectively. Previous studies have also found this to be true. In a cross-sectional survey by Li W et al. [13], it was found that MetS based on the National Cholesterol Education Program Adult Treatment Panel III (NCEP ATP III) [18] was associated with an increased risk of CVD, stroke, and HD in the middle-aged and elderly population in China by 40%, 23%, and 56%, respectively; by 34%, 20%, and 51% according to the American Heart Association/National Heart, Lung, and Blood Institute's criteria [17]; and by 31%, 24%, and 41% according to the IDF's criteria. The reason for this discrepancy may be due to the different component cut points for the different definitions. The cut points for waist circumference in NCEP ATP III and IDF are 102 cm and 90 cm for males and 88 cm and 80 cm for females, respectively. Whereas the cut points for waist circumference are the same for CDS and WCGH, 90 cm for males and 85 cm for females. The criteria for hyperglycemia in NCEP ATP III and IDF were FPG ≥ 5.6 mmol/L, whereas in CDS and WCGH it was FPG ≥ 6.1 mmol/L or 2 h PG ≥ 7.8 mmol/L. It should be emphasized that the definitions of MetS in CDS and WCGH are based on the obesity and metabolic characteristics of the Chinese population, which may be more applicable to the Chinese population. The waist circumference cutoff value for abdominal obesity in our study originates from the criteria for central obesity outlined in the “Chinese Health Industry Standard: Classification of Adult Body Weight (WS/T 428–2013)” issued by the National Health and Family Planning Commission of China in 2013 [28]. This waist circumference cutoff is widely used in large-scale epidemiological studies [29], and recent research indicates it effectively predicts CVD and all-cause mortality risk in middle-aged and older adults aged 40 years and above in mainland China [30].

The associations between the components of MetS and CVD in our study were similarly highly variable. In both definitions, the HR of high BP with CVD, stroke, and HD was the largest, with high TG and low HDL-c having the smallest associations with the three outcome events. The predominant role of hypertension aligns with previous findings in patients with type 2 diabetes in Taiwan, China, which found hypertension was the strongest component of MetS associated with ischemic heart disease [16]. This consistency across two distinct Chinese populations reinforces evidence that hypertension may be a particularly critical driver of cardiovascular risk in Chinese populations. In the study by Li W et al. [13], the association between low HDL-c and HD was the strongest; however, the association between low HDL-c and HD in our study was not significant. Furthermore, Park K et al. found that low HDL-c was not associated with asymptomatic lacunar cerebral infarction [31]. This may be related to the different cut points of HDL-c, and our study used a lower cut point. Studies have shown an “L” shaped association between HDL-c and CVD [32, 33], and the difference in the risk of CVD between the two sides of the cut-off point may become insignificant when a lower cut-off point for HDL-c is used. It may also be related to the difference in study design, as Li W et al. conducted a cross-sectional study, whereas we used a cohort study.

In the assessment and management of MetS, there is often a preference for using a simple dichotomous classification of the population as having or not having MetS. However, our research confirms that using this traditional dichotomous classification is unreasonable, as it obscures important information: participants diagnosed with MetS may face differing risks of CVD. Similar results were obtained in a cohort study by Lin C-S et al. [34], who found differences in the association between different subtypes of MetS and CVD using NCEP ATP III-based definitions. The difference was that the most significant subtype of association with CVD in the study by Lin C-S et al. was AO + BP + HDL-c, whereas in our study it was AO + GLU + BP + HDL-c and AO + GLU + BP + dyslipidemia. Unfortunately, there are still very few studies on the associations between MetS and CVD targeting different subtypes.

Although classifying MetS into subtypes would facilitate its individualized management, this is sometimes difficult to implement because of the complex diagnostic criteria for MetS that result in the existence of too many subtypes. Recommended diagnostic criteria for MetS by both NCEP-ATP III and CDS are “meets 3 of the 5 components”, and they can be categorized into 16 subtypes based on combinations of components, which makes it inconvenient to use such a large number of subtypes for disease management. Some researchers have typed the MetS according to the number of components that fulfill the MetS components [35–38], and found that the risk of CVD increases with the number of components. However, this still seems to be a rather crude approach to typing, as it still does not reflect the importance of the different components.

Our study provides new ideas for the typing and individualized management of MetS. The WCGH definition's approach of combining TG and HDL-C into a single “dyslipidemia” component appears to be a clinically judicious simplification. This strategy effectively avoids overclassifying individuals with isolated dyslipidemia as high-risk. At the same time, it successfully captures the synergistic harmful effects that occur when dyslipidemia coexists with other components, such as AO, high GLU, or high BP.

Our findings confirm that not all metabolic abnormalities carry equal CVD risk. Increased blood pressure and hyperglycemia more directly induce vascular endothelial dysfunction and proinflammatory states, both potent drivers of atherosclerosis and thrombotic events [39–41]. In contrast, our study found a relatively weak association between isolated dyslipidemia (high TG and low HDL-c) and CVD. This finding is inconsistent with earlier studies in Taiwanese populations, which indicated that high TG is a highly significant and independent predictor of CVD [42, 43]. This discrepancy may stem from methodological differences. Our study population represented a broader community cohort, and CVD assessment relied on participant self-reporting.

Among the participants we included, the prevalence of kidney disease was lower in patients with MetS than in those without MetS, which is inconsistent with findings from previous studies. It is well established that MetS and all its components are risk factors for kidney disease [44], a point particularly confirmed in populations from Taiwan, China [45, 46]. This phenomenon likely reflects underlying selection and diagnostic biases. Over 80% of participants in our study were from rural areas, with kidney disease status based on self-reports from 2011. Given the primary healthcare conditions and health screening coverage at that time, some individuals with early- or mid-stage chronic kidney disease in the community may have remained undiagnosed. Conversely, participants who were already diagnosed and aware of their kidney disease status were more likely to actively seek medical care and receive treatment for risk factors such as hypertension and diabetes. This improved their metabolic indicators, potentially rendering them no longer fully meeting the diagnostic criteria for MetS. This ultimately resulted in the lower self-reported kidney disease rate observed in the MetS group in the baseline data.

This is the first study to compare the differences between 2 Chinese-specific definitions of MetS, and innovatively analyze the differences between MetS subtypes using different typing methods. We used the nationally representative CHARLS database, which allows the findings to be generalized to a wide range of Chinese middle-aged and older community populations. Moreover, we used a large sample size and a long follow-up period, which makes the study results have good reliability. However, there are some limitations to this study. First, the study population comprised middle-aged and older adults aged 40 years and above from mainland China. Considering that diabetes, one of the most critical components of MetS and a risk factor for CVD, has been increasing among younger generations, which is mainly due to obesity, further in-depth research is needed to determine whether the findings of this study apply to younger generations [47]. Second, we used a Chinese-specific definition of MetS, and the association between subtypes of MetS based on other definitions and CVD requires further study. Third, information on participants' CVD was based on questionnaires rather than medical records, which may have led to a degree of bias.

## Conclusions

Our research suggests that the association between MetS and CVD depends on the definition criteria and specific component combinations employed. The WCGH definition, which integrates diagnostic criteria for dyslipidemia, has been demonstrated to be more robust than the CDS.

## Supplementary Information

Additional file1

## Data Availability

The data used in our study comes from China Health and Retirement Longitudinal Study (CHARLS), a publicly available database. This data can be found here: https://charls.pku.edu.cn/.

## Abbreviations

CVD: Cardiovascular Disease
MetS: Metabolic syndrome
CDS: Chinese diabetes society
WCGH: Writing group of 2024 chinese guidelines for the management of hypertension
CHARLS: China health and retirement longitudinal study
AO: Abdominal obesity
GLU: Glucose
BP: Blood pressure
TG: Triglycerides
HDL-c: High density lipoprotein cholesterol
HR: Hazard ratio
CI: Confidence interval
FPG: Fasting plasma glucose
PG: Plasma glucose
OGTT: Oral glucose tolerance test
BMI: Body mass index
HBA1c: Glycosylated hemoglobin
SBP: Systolic blood pressure
DBP: Diastolic blood pressure
CHO: Cholesterol
LDL-c: Low density lipoprotein cholesterol
IDF: International diabetes federation
NCEP ATP III: National cholesterol education program adult treatment Panel III
