# Supplementary material for: Middle-aged and older populations with different subtypes and definitions of metabolic syndrome face different future cardiovascular disease risks: results from a comparison of two Chinese definitions
Source: Nutr Metab (Lond). 2026 Feb 18;23:37. doi: 10.1186/s12986-026-01095-3 (PMC13020297; doi:10.1186/s12986-026-01095-3)
Supplement: Supplementary file 1 — Additional file1 [file 12986_2026_1095_MOESM1_ESM.docx]

**Table S1. Results of univariate Cox models of MetS and its components with CVD, stroke, and HD. (CDS)**

|  | **CVD** |  |  |  | **Stroke** |  |  |  | **HD** |  |  |
| --- | --- | --- | --- | --- | --- | --- | --- | --- | --- | --- | --- |
|  | **Cases (%)** | **HR (95 % CI)** | **P** |  | **Cases (%)** | **HR (95 % CI)** | **P** |  | **Cases (%)** | **HR (95 % CI)** | **P** |
| AO | 948 (34.6) | 1.6 (1.46~1.75) | **<0.001** |  | 330 (12) | 1.57 (1.35~1.83) | **<0.001** |  | 723 (26.4) | 1.54 (1.4~1.71) | **<0.001** |
| High GLU | 702 (34) | 1.39 (1.27~1.53) | **<0.001** |  | 267 (12.9) | 1.61 (1.38~1.87) | **<0.001** |  | 526 (25.5) | 1.32 (1.19~1.47) | **<0.001** |
| High BP | 1,262 (35.6) | 1.92 (1.75~2.1) | **<0.001** |  | 474 (13.4) | 2.37 (2.02~2.79) | **<0.001** |  | 952 (26.8) | 1.79 (1.61~1.98) | **<0.001** |
| High TG | 544 (32.2) | 1.26 (1.14~1.39) | **<0.001** |  | 200 (11.8) | 1.37 (1.17~1.62) | **<0.001** |  | 405 (24) | 1.19 (1.06~1.33) | **0.002** |
| Low HDL-c | 478 (30.3) | 1.14 (1.03~1.26) | **0.015** |  | 176 (11.2) | 1.25 (1.05~1.48) | **0.011** |  | 356 (22.6) | 1.08 (0.96~1.22) | 0.192 |
| Dyslipidemia | 869 (32.9) | 1.36 (1.25~1.49) | **<0.001** |  | 320 (12.1) | 1.52 (1.31~1.76) | **<0.001** |  | 658 (24.9) | 1.31 (1.19~1.45) | **<0.001** |
| MetS | 680 (37.1) | 1.62 (1.48~1.77) | **<0.001** |  | 255 (13.9) | 1.8 (1.55~2.1) | **<0.001** |  | 506 (27.6) | 1.5 (1.35~1.67) | **<0.001** |

MetS, metabolic syndrome; CVD, cardiovascular disease; HD, heart disease; HR, hazard ratio; CI, confidence interval; AO, abdominal obesity; GLU, glucose; BP, blood pressure; TG, triglycerides; HDL-c, high density lipoprotein cholesterol.

**Table S2. Results of univariate Cox models of subtypes of MetS with CVD, stroke, and HD. (CDS)**

| **subtypes** | **CVD** |  |  |  | **Stroke** |  |  |  | **HD** |  |  |
| --- | --- | --- | --- | --- | --- | --- | --- | --- | --- | --- | --- |
|  | **Cases (%)** | **HR (95 % CI)** | **P** |  | **Cases (%)** | **HR (95 % CI)** | **P** |  | **Cases (%)** | **HR (95 % CI)** | **P** |
| **Typing methods 1** |  |  |  |  |  |  |  |  |  |  |  |
| No MetS | 1,435 (25) | 1(Ref) |  |  | 467 (8.1) | 1(Ref) |  |  | 1,120 (19.5) | 1(Ref) |  |
| MetS A+G+B | 133 (43) | 1.99 (1.67~2.38) | **<0.001** |  | 50 (16.2) | 2.15 (1.61~2.88) | **<0.001** |  | 105 (34) | 1.97 (1.61~2.4) | **<0.001** |
| MetS A+G+T | 11 (26.2) | 1.02 (0.56~1.84) | 0.958 |  | 1 (2.4) | 0.29 (0.04~2.05) | 0.214 |  | 10 (23.8) | 1.18 (0.63~2.19) | 0.608 |
| MetS A+G+H | 13 (35.1) | 1.54 (0.89~2.65) | 0.124 |  | 5 (13.5) | 1.79 (0.74~4.32) | 0.195 |  | 9 (24.3) | 1.29 (0.67~2.48) | 0.451 |
| MetS A+B+T | 58 (40.6) | 1.83 (1.41~2.38) | **<0.001** |  | 22 (15.4) | 2 (1.3~3.07) | **0.001** |  | 40 (28) | 1.53 (1.12~2.1) | **0.008** |
| MetS A+B+H | 45 (37.5) | 1.62 (1.21~2.18) | **0.001** |  | 20 (16.7) | 2.17 (1.39~3.39) | **0.001** |  | 28 (23.3) | 1.25 (0.86~1.82) | 0.238 |
| MetS A+T+H | 21 (19.6) | 0.76 (0.49~1.17) | 0.211 |  | 3 (2.8) | 0.34 (0.11~1.05) | 0.06 |  | 19 (17.8) | 0.89 (0.56~1.4) | 0.606 |
| MetS G+B+T | 40 (41.2) | 1.84 (1.34~2.52) | **<0.001** |  | 21 (21.6) | 2.96 (1.91~4.59) | **<0.001** |  | 25 (25.8) | 1.37 (0.92~2.04) | 0.118 |
| MetS G+B+H | 15 (34.1) | 1.41 (0.85~2.35) | 0.181 |  | 5 (11.4) | 1.45 (0.6~3.51) | 0.405 |  | 11 (25) | 1.36 (0.75~2.46) | 0.312 |
| MetS G+T+H | 34 (28.1) | 1.16 (0.82~1.63) | 0.396 |  | 12 (9.9) | 1.22 (0.69~2.17) | 0.493 |  | 25 (20.7) | 1.08 (0.73~1.61) | 0.692 |
| MetS B+T+H | 20 (25.6) | 1.04 (0.67~1.61) | 0.876 |  | 6 (7.7) | 0.98 (0.44~2.19) | 0.96 |  | 16 (20.5) | 1.05 (0.64~1.72) | 0.848 |
| MetS A+G+B+T | 64 (44.1) | 2.03 (1.58~2.61) | **<0.001** |  | 25 (17.2) | 2.25 (1.51~3.37) | **<0.001** |  | 49 (33.8) | 1.93 (1.45~2.57) | **<0.001** |
| MetS A+G+B+H | 27 (45) | 2.06 (1.41~3.01) | **<0.001** |  | 10 (16.7) | 2.31 (1.24~4.32) | **0.009** |  | 23 (38.3) | 2.21 (1.46~3.34) | **<0.001** |
| MetS A+G+T+H | 25 (29.4) | 1.2 (0.81~1.78) | 0.364 |  | 8 (9.4) | 1.23 (0.61~2.47) | 0.565 |  | 19 (22.4) | 1.15 (0.73~1.81) | 0.544 |
| MetS A+B+T+H | 49 (36) | 1.54 (1.16~2.05) | **0.003** |  | 17 (12.5) | 1.58 (0.98~2.57) | 0.063 |  | 37 (27.2) | 1.46 (1.05~2.02) | **0.024** |
| MetS G+B+T+H | 38 (37.6) | 1.64 (1.19~2.27) | **0.002** |  | 14 (13.9) | 1.79 (1.05~3.04) | **0.032** |  | 32 (31.7) | 1.74 (1.23~2.48) | **0.002** |
| MetS A+G+B+T+H | 87 (42) | 1.9 (1.53~2.36) | **<0.001** |  | 36 (17.4) | 2.28 (1.62~3.2) | **<0.001** |  | 58 (28) | 1.57 (1.2~2.04) | **0.001** |
| **Typing methods 2** |  |  |  |  |  |  |  |  |  |  |  |
| No MetS | 1,435 (25) | 1(Ref) |  |  | 467 (8.1) | 1(Ref) |  |  | 1,120 (19.5) | 1(Ref) |  |
| MetS 3-components | 390 (35.5) | 1.53 (1.37~1.72) | **<0.001** |  | 145 (13.2) | 1.7 (1.41~2.05) | **<0.001** |  | 288 (26.2) | 1.42 (1.24~1.61) | **<0.001** |
| MetS 4-components | 203 (38.5) | 1.69 (1.46~1.95) | **<0.001** |  | 74 (14) | 1.83 (1.43~2.33) | **<0.001** |  | 160 (30.4) | 1.67 (1.41~1.97) | **<0.001** |
| MetS 5-components | 87 (42) | 1.9 (1.53~2.36) | **<0.001** |  | 36 (17.4) | 2.28 (1.62~3.2) | **<0.001** |  | 58 (28) | 1.57 (1.2~2.04) | **0.001** |
| **Typing methods 3** |  |  |  |  |  |  |  |  |  |  |  |
| No MetS | 1,435 (25) | 1(Ref) |  |  | 467 (8.1) | 1(Ref) |  |  | 1,120 (19.5) | 1(Ref) |  |
| MetS 3-components without T or H | 315 (39.8) | 1.77 (1.57~2) | **<0.001** |  | 124 (15.7) | 2.06 (1.69~2.51) | **<0.001** |  | 228 (28.8) | 1.59 (1.38~1.83) | **<0.001** |
| MetS 3-components with T+H | 75 (24.5) | 0.98 (0.78~1.24) | 0.885 |  | 21 (6.9) | 0.85 (0.55~1.31) | 0.451 |  | 60 (19.6) | 1 (0.77~1.3) | 0.972 |
| MetS 4-components without T or H | 91 (44.4) | 2.04 (1.65~2.52) | **<0.001** |  | 35 (17.1) | 2.27 (1.61~3.2) | **<0.001** |  | 72 (35.1) | 2.01 (1.59~2.56) | **<0.001** |
| MetS 4-components with T+H | 112 (34.8) | 1.48 (1.22~1.79) | **<0.001** |  | 39 (12.1) | 1.55 (1.12~2.16) | **0.008** |  | 88 (27.3) | 1.46 (1.18~1.82) | **0.001** |
| MetS 5-components | 87 (42) | 1.9 (1.53~2.36) | **<0.001** |  | 36 (17.4) | 2.28 (1.62~3.2) | **<0.001** |  | 58 (28) | 1.57 (1.2~2.04) | **0.001** |

MetS, metabolic syndrome; CVD, cardiovascular disease; HD, heart disease; HR, hazard ratio; CI, confidence interval; A, abdominal obesity; G, high glucose; B, high blood pressure; T, high triglycerides; H, low high density lipoprotein cholesterol.

**Table S3. Results of inverse probability weighted multifactorial Cox models of MetS and its components with CVD, stroke, and HD. (CDS)**

|  | **CVD** |  |  |  | **Stroke** |  |  |  | **HD** |  |  |
| --- | --- | --- | --- | --- | --- | --- | --- | --- | --- | --- | --- |
|  | **Cases (%)** | **HR (95 % CI)** | **P** |  | **Cases (%)** | **HR (95 % CI)** | **P** |  | **Cases (%)** | **HR (95 % CI)** | **P** |
| AO | 948 (34.6) | 1.535 (1.394~1.69) | **<0.001** |  | 330 (12) | 1.669 (1.409~1.976) | **<0.001** |  | 723 (26.4) | 1.437 (1.289~1.603) | **<0.001** |
| High GLU | 702 (34) | 1.306 (1.189~1.434) | **<0.001** |  | 267 (12.9) | 1.457 (1.242~1.708) | **<0.001** |  | 526 (25.5) | 1.261 (1.132~1.405) | **<0.001** |
| High BP | 1,262 (35.6) | 1.715 (1.559~1.887) | **<0.001** |  | 474 (13.4) | 2.062 (1.739~2.445) | **<0.001** |  | 952 (26.8) | 1.634 (1.465~1.823) | **<0.001** |
| High TG | 544 (32.2) | 1.237 (1.118~1.369) | **<0.001** |  | 200 (11.8) | 1.39 (1.171~1.649) | **<0.001** |  | 405 (24) | 1.158 (1.03~1.301) | **0.014** |
| Low HDL-c | 478 (30.3) | 1.14 (1.025~1.267) | **0.016** |  | 176 (11.2) | 1.184 (0.99~1.415) | 0.064 |  | 356 (22.6) | 1.108 (0.98~1.251) | 0.101 |
| Dyslipidemia | 869 (32.9) | 1.325 (1.21~1.451) | **<0.001** |  | 320 (12.1) | 1.474 (1.262~1.721) | **<0.001** |  | 658 (24.9) | 1.272 (1.146~1.411) | **<0.001** |
| MetS | 680 (37.1) | 1.459 (1.325~1.607) | **<0.001** |  | 255 (13.9) | 1.633 (1.388~1.922) | **<0.001** |  | 506 (27.6) | 1.373 (1.23~1.534) | **<0.001** |

MetS, metabolic syndrome; CVD, cardiovascular disease; HD, heart disease; HR, hazard ratio; CI, confidence interval; AO, abdominal obesity; GLU, glucose; BP, blood pressure; TG, triglycerides; HDL-c, high density lipoprotein cholesterol.

**Table S4. Results of inverse probability weighted multifactorial Cox models of subtypes of MetS with CVD, stroke, and HD. (CDS)**

| **subtypes** | **CVD** |  |  |  | **Stroke** |  |  |  | **HD** |  |  |
| --- | --- | --- | --- | --- | --- | --- | --- | --- | --- | --- | --- |
|  | **Cases (%)** | **HR (95 % CI)** | **P** |  | **Cases (%)** | **HR (95 % CI)** | **P** |  | **Cases (%)** | **HR (95 % CI)** | **P** |
| **Typing methods 1** |  |  |  |  |  |  |  |  |  |  |  |
| No MetS | 1,435 (25) | 1(Ref) |  |  | 467 (8.1) | 1(Ref) |  |  | 1,120 (19.5) | 1(Ref) |  |
| MetS A+G+B | 133 (43) | 1.59 (1.302~1.943) | **<0.001** |  | 50 (16.2) | 1.694 (1.235~2.325) | **0.001** |  | 105 (34) | 1.612 (1.293~2.011) | **<0.001** |
| MetS A+G+T | 11 (26.2) | 0.836 (0.447~1.563) | 0.575 |  | 1 (2.4) | 0.251 (0.034~1.861) | 0.176 |  | 10 (23.8) | 0.934 (0.489~1.785) | 0.837 |
| MetS A+G+H | 13 (35.1) | 1.495 (0.862~2.593) | 0.153 |  | 5 (13.5) | 1.824 (0.764~4.353) | 0.176 |  | 9 (24.3) | 1.198 (0.607~2.365) | 0.603 |
| MetS A+B+T | 58 (40.6) | 1.684 (1.293~2.193) | **<0.001** |  | 22 (15.4) | 1.974 (1.272~3.063) | **0.002** |  | 40 (28) | 1.38 (0.999~1.907) | 0.051 |
| MetS A+B+H | 45 (37.5) | 1.412 (1.03~1.936) | **0.032** |  | 20 (16.7) | 1.609 (0.997~2.598) | 0.051 |  | 28 (23.3) | 1.199 (0.811~1.772) | 0.363 |
| MetS A+T+H | 21 (19.6) | 0.837 (0.539~1.3) | 0.429 |  | 3 (2.8) | 0.354 (0.114~1.099) | 0.072 |  | 19 (17.8) | 0.957 (0.602~1.519) | 0.851 |
| MetS G+B+T | 40 (41.2) | 1.519 (1.107~2.083) | **0.01** |  | 21 (21.6) | 2.418 (1.534~3.812) | **<0.001** |  | 25 (25.8) | 1.226 (0.836~1.798) | 0.297 |
| MetS G+B+H | 15 (34.1) | 1.083 (0.671~1.747) | 0.744 |  | 5 (11.4) | 0.938 (0.363~2.424) | 0.895 |  | 11 (25) | 1.14 (0.664~1.96) | 0.634 |
| MetS G+T+H | 34 (28.1) | 1.244 (0.864~1.792) | 0.24 |  | 12 (9.9) | 1.265 (0.674~2.374) | 0.465 |  | 25 (20.7) | 1.267 (0.836~1.921) | 0.265 |
| MetS B+T+H | 20 (25.6) | 0.922 (0.569~1.495) | 0.743 |  | 6 (7.7) | 0.83 (0.358~1.923) | 0.664 |  | 16 (20.5) | 0.966 (0.562~1.661) | 0.901 |
| MetS A+G+B+T | 64 (44.1) | 1.886 (1.492~2.385) | **<0.001** |  | 25 (17.2) | 2.27 (1.507~3.419) | **<0.001** |  | 49 (33.8) | 1.716 (1.296~2.273) | **<0.001** |
| MetS A+G+B+H | 27 (45) | 1.808 (1.214~2.691) | **0.004** |  | 10 (16.7) | 2.185 (1.134~4.21) | **0.02** |  | 23 (38.3) | 1.999 (1.306~3.062) | **0.001** |
| MetS A+G+T+H | 25 (29.4) | 1.207 (0.813~1.791) | 0.35 |  | 8 (9.4) | 1.348 (0.655~2.772) | 0.417 |  | 19 (22.4) | 1.143 (0.72~1.815) | 0.572 |
| MetS A+B+T+H | 49 (36) | 1.431 (1.07~1.914) | **0.016** |  | 17 (12.5) | 1.554 (0.955~2.529) | 0.076 |  | 37 (27.2) | 1.318 (0.935~1.86) | 0.115 |
| MetS G+B+T+H | 38 (37.6) | 1.512 (1.086~2.106) | **0.014** |  | 14 (13.9) | 1.51 (0.862~2.645) | 0.15 |  | 32 (31.7) | 1.629 (1.145~2.318) | **0.007** |
| MetS A+G+B+T+H | 87 (42) | 1.708 (1.355~2.153) | **<0.001** |  | 36 (17.4) | 2.091 (1.46~2.995) | **<0.001** |  | 58 (28) | 1.371 (1.029~1.826) | **0.031** |
| **Typing methods 2** |  |  |  |  |  |  |  |  |  |  |  |
| No MetS | 1,435 (25) | 1(Ref) |  |  | 467 (8.1) | 1(Ref) |  |  | 1,120 (19.5) | 1(Ref) |  |
| MetS 3-components | 390 (35.5) | 1.365 (1.212~1.537) | **<0.001** |  | 145 (13.2) | 1.482 (1.216~1.807) | **<0.001** |  | 288 (26.2) | 1.3 (1.135~1.49) | **<0.001** |
| MetS 4-components | 203 (38.5) | 1.571 (1.354~1.823) | **<0.001** |  | 74 (14) | 1.781 (1.38~2.3) | **<0.001** |  | 160 (30.4) | 1.532 (1.293~1.816) | **<0.001** |
| MetS 5-components | 87 (42) | 1.703 (1.351~2.148) | **<0.001** |  | 36 (17.4) | 2.087 (1.456~2.991) | **<0.001** |  | 58 (28) | 1.367 (1.026~1.821) | **0.033** |
| **Typing methods 3** |  |  |  |  |  |  |  |  |  |  |  |
| No MetS | 1,435 (25) | 1(Ref) |  |  | 467 (8.1) | 1(Ref) |  |  | 1,120 (19.5) | 1(Ref) |  |
| MetS 3-components without T or H | 315 (39.8) | 1.486 (1.304~1.694) | **<0.001** |  | 124 (15.7) | 1.692 (1.366~2.095) | **<0.001** |  | 228 (28.8) | 1.378 (1.186~1.602) | **<0.001** |
| MetS 3-components with T+H | 75 (24.5) | 1.007 (0.786~1.291) | 0.955 |  | 21 (6.9) | 0.844 (0.528~1.351) | 0.480 |  | 60 (19.6) | 1.067 (0.811~1.406) | 0.642 |
| MetS 4-components without T or H | 91 (44.4) | 1.857 (1.511~2.284) | **<0.001** |  | 35 (17.1) | 2.238 (1.571~3.188) | **<0.001** |  | 72 (35.1) | 1.799 (1.417~2.283) | **<0.001** |
| MetS 4-components with T+H | 112 (34.8) | 1.4 (1.151~1.705) | **0.001** |  | 39 (12.1) | 1.494 (1.065~2.096) | **0.02** |  | 88 (27.3) | 1.372 (1.097~1.715) | **0.006** |
| MetS 5-components | 87 (42) | 1.707 (1.354~2.151) | **<0.001** |  | 36 (17.4) | 2.091 (1.46~2.994) | **<0.001** |  | 58 (28) | 1.369 (1.028~1.823) | 0.032 |

MetS, metabolic syndrome; CVD, cardiovascular disease; HD, heart disease; HR, hazard ratio; CI, confidence interval; A, abdominal obesity; G, high glucose; B, high blood pressure; T, high triglycerides; H, low high density lipoprotein cholesterol.

**Table S5. Results of univariate Cox models of MetS and its components with CVD, stroke, and HD. (WCGH)**

|  | **CVD** |  |  |  | **Stroke** |  |  |  | **HD** |  |  |
| --- | --- | --- | --- | --- | --- | --- | --- | --- | --- | --- | --- |
|  | **Cases (%)** | **HR (95 % CI)** | **P** |  | **Cases (%)** | **HR (95 % CI)** | **P** |  | **Cases (%)** | **HR (95 % CI)** | **P** |
| AO | 980 (35) | 1.74 (1.6~1.9) | **<0.001** |  | 339 (12.1) | 1.73 (1.5~2.01) | **<0.001** |  | 749 (26.7) | 1.69 (1.53~1.86) | **<0.001** |
| High GLU | 697 (34.1) | 1.4 (1.28~1.53) | **<0.001** |  | 261 (12.8) | 1.58 (1.36~1.84) | **<0.001** |  | 524 (25.6) | 1.33 (1.2~1.48) | **<0.001** |
| High BP | 1,289 (36) | 2.06 (1.9~2.25) | **<0.001** |  | 477 (13.3) | 2.5 (2.15~2.91) | **<0.001** |  | 977 (27.3) | 1.95 (1.77~2.15) | **<0.001** |
| High TG | 523 (32.2) | 1.26 (1.14~1.39) | **<0.001** |  | 190 (11.7) | 1.36 (1.15~1.6) | **<0.001** |  | 392 (24.2) | 1.2 (1.07~1.35) | **0.002** |
| Low HDL-c | 457 (30.2) | 1.13 (1.02~1.26) | **0.02** |  | 166 (11) | 1.22 (1.03~1.46) | **0.023** |  | 343 (22.7) | 1.09 (0.97~1.23) | 0.169 |
| Dyslipidemia | 881 (33.1) | 1.37 (1.26~1.5) | **<0.001** |  | 315 (11.8) | 1.48 (1.27~1.71) | **<0.001** |  | 674 (25.3) | 1.34 (1.21~1.48) | **<0.001** |
| MetS | 697 (40.8) | 2 (1.83~2.18) | **<0.001** |  | 260 (15.2) | 2.22 (1.91~2.58) | **<0.001** |  | 524 (30.7) | 1.87 (1.68~2.07) | **<0.001** |

MetS, metabolic syndrome; CVD, cardiovascular disease; HD, heart disease; HR, hazard ratio; CI, confidence interval; AO, abdominal obesity; GLU, glucose; BP, blood pressure; TG, triglycerides; HDL-c, high density lipoprotein cholesterol.

**Table S6. Results of univariate Cox models of subtypes of MetS with CVD, stroke, and HD. (WCGH)**

| **subtypes** | **CVD** |  |  |  | **Stroke** |  |  |  | **HD** |  |  |
| --- | --- | --- | --- | --- | --- | --- | --- | --- | --- | --- | --- |
|  | **Cases (%)** | **HR (95 % CI)** | **P** |  | **Cases (%)** | **HR (95 % CI)** | **P** |  | **Cases (%)** | **HR (95 % CI)** | **P** |
| No MetS | 1,573 (23.2) | 1(Ref) |  |  | 498 (7.3) | 1(Ref) |  |  | 1,226 (18.1) | 1(Ref) |  |
| MetS A+G+B | 103 (40.6) | 2.01 (1.65~2.45) | **<0.001** |  | 38 (15) | 2.21 (1.59~3.07) | **<0.001** |  | 80 (31.5) | 1.96 (1.56~2.46) | **<0.001** |
| MetS A+G+D | 61 (32.1) | 1.45 (1.13~1.88) | **0.004** |  | 20 (10.5) | 1.51 (0.96~2.35) | 0.073 |  | 48 (25.3) | 1.43 (1.07~1.9) | **0.016** |
| MetS A+B+D | 216 (41.7) | 2.05 (1.77~2.36) | **<0.001** |  | 73 (14.1) | 2.02 (1.58~2.58) | **<0.001** |  | 161 (31.1) | 1.89 (1.6~2.23) | **<0.001** |
| MetS G+B+D | 109 (39.2) | 1.89 (1.56~2.3) | **<0.001** |  | 46 (16.5) | 2.43 (1.8~3.29) | **<0.001** |  | 80 (28.8) | 1.72 (1.37~2.15) | **<0.001** |
| MetS A+G+B+D | 208 (44.5) | 2.25 (1.95~2.6) | **<0.001** |  | 83 (17.8) | 2.62 (2.08~3.31) | **<0.001** |  | 155 (33.2) | 2.08 (1.76~2.45) | **<0.001** |

MetS, metabolic syndrome; CVD, cardiovascular disease; HD, heart disease; HR, hazard ratio; CI, confidence interval; A, abdominal obesity; G, high glucose; B, high blood pressure; D, dyslipidemia.

**Table S7. Results of inverse probability weighted multifactorial Cox models of MetS and its components with CVD, stroke, and HD. (WCGH)**

|  | **CVD** |  |  |  | **Stroke** |  |  |  | **HD** |  |  |
| --- | --- | --- | --- | --- | --- | --- | --- | --- | --- | --- | --- |
|  | **Cases (%)** | **HR (95 % CI)** | **P** |  | **Cases (%)** | **HR (95 % CI)** | **P** |  | **Cases (%)** | **HR (95 % CI)** | **P** |
| AO | 980 (35) | 1.626 (1.481~1.784) | **<0.001** |  | 339 (12.1) | 1.768 (1.5~2.085) | **<0.001** |  | 749 (26.7) | 1.528 (1.375~1.698) | **<0.001** |
| High GLU | 697 (34.1) | 1.298 (1.18~1.427) | **<0.001** |  | 261 (12.8) | 1.425 (1.212~1.675) | **<0.001** |  | 524 (25.6) | 1.256 (1.127~1.401) | **<0.001** |
| High BP | 1,289 (36) | 1.822 (1.664~1.994) | **<0.001** |  | 477 (13.3) | 2.122 (1.805~2.496) | **<0.001** |  | 977 (27.3) | 1.757 (1.584~1.949) | **<0.001** |
| High TG | 523 (32.2) | 1.236 (1.115~1.369) | **<0.001** |  | 190 (11.7) | 1.386 (1.164~1.651) | **<0.001** |  | 392 (24.2) | 1.158 (1.028~1.304) | **0.015** |
| Low HDL-c | 457 (30.2) | 1.125 (1.010~1.253) | **0.032** |  | 166 (11) | 1.157 (0.964~1.388) | 0.118 |  | 343 (22.7) | 1.098 (0.969~1.243) | 0.142 |
| Dyslipidemia | 881 (33.1) | 1.324 (1.209~1.45) | **<0.001** |  | 315 (11.8) | 1.428 (1.22~1.671) | **<0.001** |  | 674 (25.3) | 1.285 (1.158~1.426) | **<0.001** |
| MetS | 697 (40.8) | 1.72 (1.563~1.893) | **<0.001** |  | 260 (15.2) | 1.924 (1.634~2.265) | **<0.001** |  | 524 (30.7) | 1.623 (1.455~1.811) | **<0.001** |

MetS, metabolic syndrome; CVD, cardiovascular disease; HD, heart disease; HR, hazard ratio; CI, confidence interval; AO, abdominal obesity; GLU, glucose; BP, blood pressure; TG, triglycerides; HDL-c, high density lipoprotein cholesterol.

**Table S8. Results of inverse probability weighted multifactorial Cox models of subtypes of MetS with CVD, stroke, and HD. (WCGH)**

| **subtypes** | **CVD** |  |  |  | **Stroke** |  |  |  | **HD** |  |  |
| --- | --- | --- | --- | --- | --- | --- | --- | --- | --- | --- | --- |
|  | **Cases (%)** | **HR (95 % CI)** | **P** |  | **Cases (%)** | **HR (95 % CI)** | **P** |  | **Cases (%)** | **HR (95 % CI)** | **P** |
| No MetS | 1,573 (23.2) | 1(Ref) |  |  | 498 (7.3) | 1(Ref) |  |  | 1,226 (18.1) | 1(Ref) |  |
| MetS A+G+B | 103 (40.6) | 1.598 (1.278~1.999) | **<0.001** |  | 38 (15) | 1.694 (1.185~2.421) | **0.004** |  | 80 (31.5) | 1.607 (1.253~2.061) | **<0.001** |
| MetS A+G+D | 61 (32.1) | 1.355 (1.04~1.764) | **0.024** |  | 20 (10.5) | 1.617 (1.012~2.584) | **0.044** |  | 48 (25.3) | 1.281 (0.95~1.729) | 0.105 |
| MetS A+B+D | 216 (41.7) | 1.793 (1.543~2.083) | **<0.001** |  | 73 (14.1) | 1.815 (1.394~2.363) | **<0.001** |  | 161 (31.1) | 1.657 (1.392~1.973) | **<0.001** |
| MetS G+B+D | 109 (39.2) | 1.582 (1.29~1.938) | **<0.001** |  | 46 (16.5) | 1.82 (1.31~2.528) | **<0.001** |  | 80 (28.8) | 1.554 (1.235~1.956) | **<0.001** |
| MetS A+G+B+D | 208 (44.5) | 1.959 (1.681~2.284) | **<0.001** |  | 83 (17.8) | 2.37 (1.85~3.034) | **<0.001** |  | 155 (33.2) | 1.784 (1.491~2.134) | **<0.001** |

MetS, metabolic syndrome; CVD, cardiovascular disease; HD, heart disease; HR, hazard ratio; CI, confidence interval; A, abdominal obesity; G, high glucose; B, high blood pressure; D, dyslipidemia.

MetS, metabolic syndrome; CVD, cardiovascular disease; HD, heart disease; HR, hazard ratio; CI, confidence interval; A, abdominal obesity; G, high glucose; B, high blood pressure; D, dyslipidemia.

**Table S9. The time dependent ROC of CVD.**

| **Definitions** | **Time (year)** | **AUC (95%CI)** |
| --- | --- | --- |
| CDS |  |  |
|  | 2 | 0.573 (0.542 ~ 0.603) |
|  | 4 | 0.554 (0.537 ~ 0.571) |
|  | 6 | 0.557 (0.543 ~ 0.571) |
|  | 8 | 0.560 (0.547 ~ 0.572) |
| WCGH |  |  |
|  | 2 | 0.583 (0.555 ~ 0.612) |
|  | 4 | 0.574 (0.558 ~ 0.590) |
|  | 6 | 0.574 (0.561 ~ 0.587) |
|  | 8 | 0.578 (0.567 ~ 0.590) |

**Table S10. Results of univariate logistic models of MetS diagnosed based on CDS and WCGH with CVD, stroke, and HD.**

| **Groups** | **CVD** |  |  |  | **Stroke** |  |  |  | **HD** |  |  |
| --- | --- | --- | --- | --- | --- | --- | --- | --- | --- | --- | --- |
|  | **Cases (%)** | **OR (95 % CI)** | **P** |  | **Cases (%)** | **OR (95 % CI)** | **P** |  | **Cases (%)** | **OR (95 % CI)** | **P** |
| CDS- WCGH- | 771 (10.4) | 1(Ref) |  |  | 114 (1.5) | 1(Ref) |  |  | 672 (9) | 1(Ref) |  |
| CDS+ WCGH- | 33 (10.9) | 1.06 (0.73~1.53) | 0.763 |  | 8 (2.6) | 1.74 (0.84~3.61) | 0.133 |  | 27 (8.9) | 0.99 (0.66~1.48) | 0.946 |
| CDS- WCGH+ | 46 (29.1) | 3.56 (2.5~5.05) | **<0.001** |  | 5 (3.2) | 2.1 (0.85~5.22) | 0.109 |  | 42 (26.6) | 3.65 (2.54~5.24) | **<0.001** |
| CDS+ WCGH+ | 544 (22.8) | 2.56 (2.26~2.89) | **<0.001** |  | 121 (5.1) | 3.44 (2.65~4.46) | **<0.001** |  | 458 (19.2) | 2.39 (2.1~2.72) | **<0.001** |

MetS, metabolic syndrome; CVD, cardiovascular disease; HD, heart disease; OR, odds ratio; CI, confidence interval.

**Table S11. Results of multifactorial logistic models of MetS diagnosed based on CDS and WCGH with CVD, stroke, and HD.**

| **Groups** | **CVD** |  |  |  | **Stroke** |  |  |  | **HD** |  |  |
| --- | --- | --- | --- | --- | --- | --- | --- | --- | --- | --- | --- |
|  | **Cases (%)** | **OR (95 % CI)** | **P** |  | **Cases (%)** | **OR (95 % CI)** | **P** |  | **Cases (%)** | **OR (95 % CI)** | **P** |
| CDS- WCGH- | 771 (10.4) | 1(Ref) |  |  | 114 (1.5) | 1(Ref) |  |  | 672 (9) | 1(Ref) |  |
| CDS+ WCGH- | 33 (10.9) | 1.11 (0.76~1.61) | 0.597 |  | 8 (2.6) | 1.87 (0.9~3.9) | 0.093 |  | 27 (8.9) | 1.02 (0.68~1.54) | 0.909 |
| CDS- WCGH+ | 46 (29.1) | 3.12 (2.17~4.5) | **<0.001** |  | 5 (3.2) | 1.98 (0.79~4.97) | 0.146 |  | 42 (26.6) | 3.14 (2.16~4.58) | **<0.001** |
| CDS+ WCGH+ | 544 (22.8) | 2.23 (1.97~2.54) | **<0.001** |  | 121 (5.1) | 3.12 (2.37~4.1) | **<0.001** |  | 458 (19.2) | 2.07 (1.8~2.37) | **<0.001** |

MetS, metabolic syndrome; CVD, cardiovascular disease; HD, heart disease; OR, odds ratio; CI, confidence interval.

Adjusted: gender, age, education, marriage, residence, smoking, alcohol intake, chronic lung disease, kidney disease.

**Table S12. Results of univariate Cox models of MetS diagnosed based on CDS and WCGH with CVD, stroke, and HD.**

| **Groups** | **CVD** |  |  |  | **Stroke** |  |  |  | **HD** |  |  |
| --- | --- | --- | --- | --- | --- | --- | --- | --- | --- | --- | --- |
|  | **Cases (%)** | **HR (95 % CI)** | **P** |  | **Cases (%)** | **HR (95 % CI)** | **P** |  | **Cases (%)** | **HR (95 % CI)** | **P** |
| CDS- WCGH- | 1364 (24.5) | 1(Ref) |  |  | 444 (8) | 1(Ref) |  |  | 1060 (19) | 1(Ref) |  |
| CDS+ WCGH- | 53 (22.3) | 0.91 (0.69~1.19) | 0.481 |  | 11 (4.6) | 0.57 (0.32~1.04) | 0.069 |  | 46 (19.3) | 1.01 (0.76~1.36) | 0.924 |
| CDS- WCGH+ | 52 (54.2) | 2.73 (2.07~3.6) | **<0.001** |  | 17 (17.7) | 2.38 (1.47~3.86) | **<0.001** |  | 45 (46.9) | 2.86 (2.12~3.86) | **<0.001** |
| CDS+ WCGH+ | 605 (39.6) | 1.8 (1.64~1.98) | **<0.001** |  | 234 (15.3) | 2.05 (1.75~2.41) | **<0.001** |  | 446 (29.2) | 1.66 (1.48~1.85) | **<0.001** |

MetS, metabolic syndrome; CVD, cardiovascular disease; HD, heart disease; HR, hazard ratio; CI, confidence interval.

**Table S13. Results of multifactorial Cox models of MetS diagnosed based on CDS and WCGH with CVD, stroke, and HD.**

| **Groups** | **CVD** |  |  |  | **Stroke** |  |  |  | **HD** |  |  |
| --- | --- | --- | --- | --- | --- | --- | --- | --- | --- | --- | --- |
|  | **Cases (%)** | **HR (95 % CI)** | **P** |  | **Cases (%)** | **HR (95 % CI)** | **P** |  | **Cases (%)** | **HR (95 % CI)** | **P** |
| CDS- WCGH- | 1364 (24.5) | 1(Ref) |  |  | 444 (8) | 1(Ref) |  |  | 1060 (19) | 1(Ref) |  |
| CDS+ WCGH- | 53 (22.3) | 0.93 (0.7~1.23) | 0.606 |  | 11 (4.6) | 0.62 (0.34~1.13) | 0.116 |  | 46 (19.3) | 1.02 (0.76~1.38) | 0.882 |
| CDS- WCGH+ | 52 (54.2) | 2.57 (1.94~3.39) | **<0.001** |  | 17 (17.7) | 2.28 (1.4~3.71) | **0.001** |  | 45 (46.9) | 2.65 (1.96~3.58) | **<0.001** |
| CDS+ WCGH+ | 605 (39.6) | 1.66 (1.51~1.83) | **<0.001** |  | 234 (15.3) | 1.95 (1.66~2.29) | **<0.001** |  | 446 (29.2) | 1.52 (1.36~1.7) | **<0.001** |

MetS, metabolic syndrome; CVD, cardiovascular disease; HD, heart disease; HR, hazard ratio; CI, confidence interval.

Adjusted: gender, age, education, marriage, residence, smoking, alcohol intake, chronic lung disease, kidney disease.

**Table S14. Results of NRI and IDI analysis of logistic model**

| logistic | **CVD** |  |  | **Stroke** |  |  | **HD** |  |
| --- | --- | --- | --- | --- | --- | --- | --- | --- |
|  | **Est. (95 % CI)** | **P** |  | **Est. (95 % CI)** | **P** |  | **Est. (95 % CI)** | **P** |
| NRI(Categorical) | 0.0352 (0.0203 ~ 0.0501) | **<0.001** |  | -0.0002 (-0.0005 ~ 0.0001) | 0.157 |  | 0.0071 ( -0.0061 ~ 0.0203) | 0.295 |
| NRI(Continuous) | 0.4050 (0.3497 ~ 0.4603) | **<0.001** |  | -0.0623 (-0.1633 ~ 0.0387) | 0.226 |  | 0.3813 (0.3223 ~ 0.4403) | **<0.001** |
| IDI | 0.0052 (0.0036 ~ 0.0069) | **<0.001** |  | 0.0003 (-0.001 ~ 0.0016) | 0.667 |  | 0.0047 (0.0032 ~ 0.0062) | **<0.001** |

NRI, net reclassification improvement; IDI, integrated discrimination improvement. CVD, cardiovascular disease; HD, heart disease; CI, confidence interval.

**Table S15. Results of NRI and IDI analysis of Cox model**

| Cox | **CVD** |  |  | **Stroke** |  |  | **HD** |  |
| --- | --- | --- | --- | --- | --- | --- | --- | --- |
|  | **Est. (95 % CI)** | **P** |  | **Est. (95 % CI)** | **P** |  | **Est. (95 % CI)** | **P** |
| NRI(Categorical) | 0.021 (0.008 ~ 0.037) | **<0.001** |  | 0.022 (-0.001 ~ 0.044) | 0.065 |  | 0.02 (0.002 ~ 0.042) | **<0.001** |
| NRI(Continuous) | 0.171 (0.109 ~ 0.192) | **<0.001** |  | 0.152 (0.095 ~ 0.184) | **0.008** |  | 0.115 (0.038 ~ 0.14) | **0.008** |
| IDI | 0.009 (0.006 ~ 0.012) | **<0.001** |  | 0.005 (0.002 ~ 0.007) | **<0.001** |  | 0.004 (0.002 ~ 0.007) | **<0.001** |

NRI, net reclassification improvement; IDI, integrated discrimination improvement. CVD, cardiovascular disease; HD, heart disease; CI, confidence interval.
